# A critical role for erythropoietin on vagus nerve Schwann cells in intestinal motility

**DOI:** 10.1186/s12896-023-00781-x

**Published:** 2023-05-01

**Authors:** Prem Kumar Govindappa, Mosammat Begom, Yash Gupta, John C. Elfar, Manmeet Rawat, Walaa Elfar

**Affiliations:** 1 Department of Orthopaedics and Sports Medicine, University of Arizona College of Medicine, Tucson, AZ 85724 USA; 2 Department of Medicine, The Penn State University College of Medicine, Hershey, PA 17033 USA; 3 Department of Pediatrics, University of Arizona College of Medicine, Tucson, AZ 85724 USA

**Keywords:** Erythropoietin, Erythropoietin receptors knockout, Structural analysis, Vagus nerve, Schwann cells, Enteric glial cells, Intestinal manipulation, Intestinal transit time, Postoperative ileus.

## Abstract

**Background:**

Dysmotility and postoperative ileus (POI) are frequent major clinical problems post-abdominal surgery. Erythropoietin (EPO) is a multifunctional tissue-protective cytokine that promotes recovery of the intestine in various injury models. While EPO receptors (EPOR) are present in vagal Schwann cells, the role of EPOR in POI recovery is unknown because of the lack of EPOR antagonists or Schwann-cell specific EPOR knockout animals. This study was designed to explore the effect of EPO via EPOR in vagal nerve Schwann cells in a mouse model of POI.

**Results:**

The structural features of EPOR and its activation by EPO-mediated dimerization were understood using structural analysis. Later, using the Cre-loxP system, we developed a myelin protein zero (Mpz) promoter-driven knockout mouse model of Schwann cell EPOR (MpzCre-EPOR^flox/flox^ / Mpz-EPOR-KO) confirmed using PCR and qRT-PCR techniques. We then measured the intestinal transit time (ITT) at baseline and after induction of POI with and without EPO treatment. Although we have previously shown that EPO accelerates functional recovery in POI in wild type mice, EPO treatment did not improve functional recovery of ITT in POI of Mpz-EPOR-KO mice.

**Conclusions:**

To the best of our knowledge, this is the first pre-clinical study to demonstrate a novel mouse model of EPOR specific knock out on Schwan cells with an effect in the gut. We also showed novel beneficial effects of EPO through vagus nerve Schwann cell-EPOR in intestinal dysmotility. Our findings suggest that EPO-EPOR signaling in the vagus nerve after POI is important for the functional recovery of ITT.

**Supplementary Information:**

The online version contains supplementary material available at 10.1186/s12896-023-00781-x.

## Background

Postoperative ileus (POI) is a common clinical complication after an abdominal surgery that involves inhibition of intestinal motility, prolonged parenteral nutrition, abdominal discomfort (e.g., pain, bloating, constipation), and increased morbidity [1, 2]. The incidence of POI was reported in various cohorts in 10 to 30% of patients after abdominal surgery [3–5]. Normal intestinal motility is critical for nutrition assimilation and several biological functions; the loss of normal gut function aggravates inflammation, oxidative stress, and other cellular stressors that prolong patient recovery and hospital stay [1, 4]. The pathophysiological mechanisms of POI are multifactorial and are associated with complex biological mediators [6, 7]. Despite the high occurrence of POI, currently, no treatment is effective against gastrointestinal tract dysmotility.

Erythropoietin (EPO) is a U.S. Food and Drug Administration (FDA) approved drug for the treatment of anemia associated with chronic kidney disease. The functional role of EPO via EPO receptors (EPOR) has been reported in many other tissues including the intestine [8–10]. Our recent study has shown a novel beneficial effect of EPO against POI by reducing oxidative stress and inflammatory insults in wild-type mice [11]. However, the mechanistic insights in EPO-EPOR mediated resolution of POI, and its therapeutic potential in the clinic need further investigations. It is known that the manipulation of the intestine triggers a neuronal reflex inhibition of intestinal motility besides cellular stress [12, 13]. However, none of the studies demonstrated the vagus nerve (VN) role in intestinal functional recovery in POI. The VN is the longest extrinsic cranial nerve in the body, regulates gut physiology through the intrinsic nervous system (myenteric and submucosal plexus) and enteric glial cells (EGCs) interactions, which participate in controlling intestinal absorption, secretion, immune homeostasis, and motility [14–16]. Our studies have shown that EPO enhances recovery after traumatic peripheral nerve injury through EPOR on Schwan cells [17–20].

Thus, we aimed to understand the obligatory role of VN Schwann-cell specific EPOR in EPO-induced intestinal functional recovery. While EPOR is present in Schwann cells of the VN, the role of EPOR in POI recovery is unknown because of the lack of EPOR antagonists or Schwann cell specific EPOR knockout animals. This study was designed to address these questions by testing a clinically relevant systemic dosage of EPO using the Mpz-EPOR-KO-in vivo murine model after inducing POI.

## Results

**Fig. 1:**
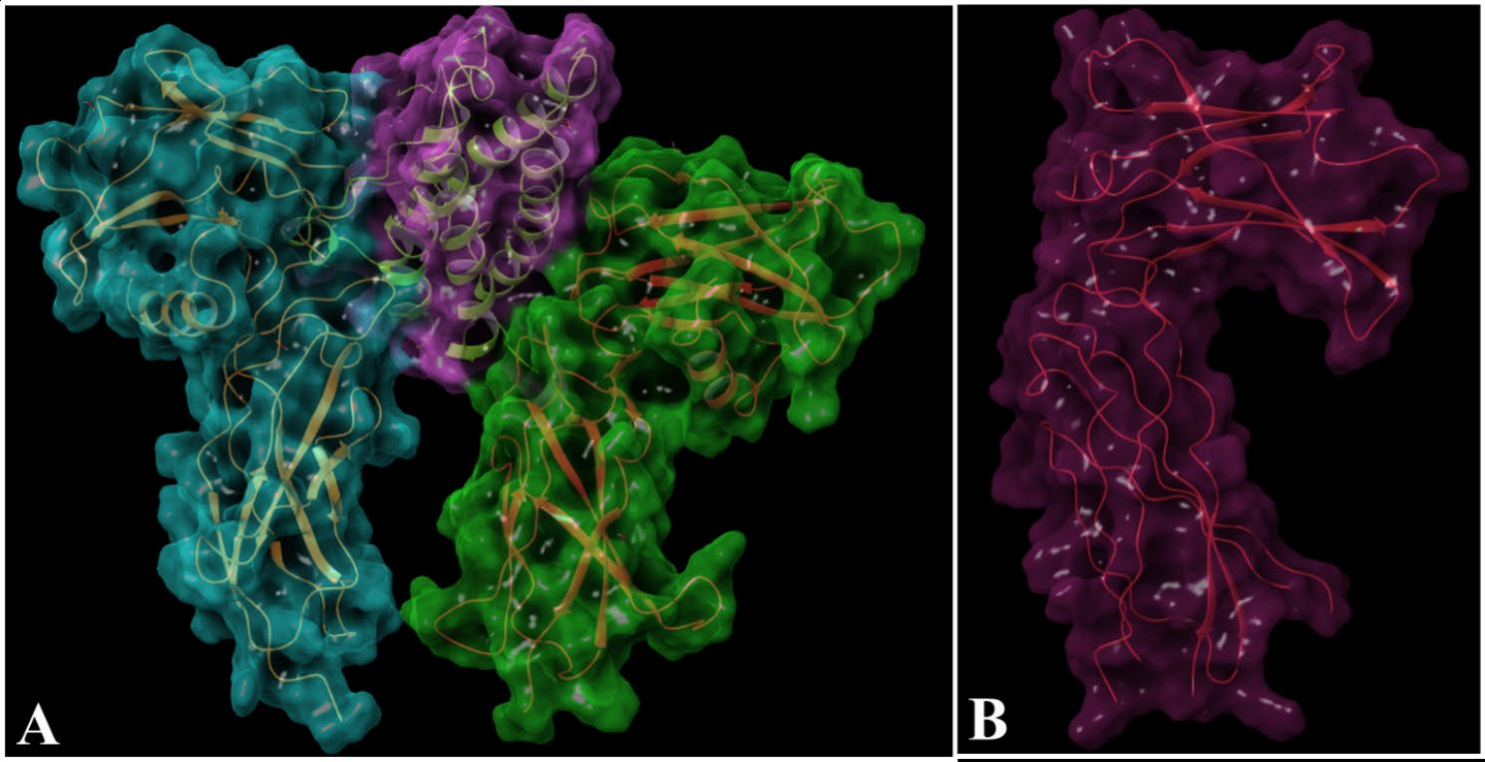
3-dimensional visual protein surface representations of the contact interfaces. **A** One view of the complex showing dimerized native structure of the extracellular domain of erythropoietin receptor (EPOR) in green and blue color in the presence of EPO (Purple color). Model shows interlocked surfaces between both the subunits of EPOR at different sites of EPO hormone (PDB ID 1CN4; 10.2210/pdb1CN4/pdb). **B** Another view of the EPOR monomer stabilized by the allosteric antagonist (PDB ID 1ERN; 10.2210/pdb1ERN/pdb). The two amino acids most critical for both the interactions are Ser91 and His153 of EPOR which are masked by the inhibitor impeding dimerization

To understand the structural features of EPOR and EPOR activation by EPO mediated dimerization, we performed a 3-dimensional bioinformatics analysis. The X-ray crystal structures of EPOR bound to EPO and antagonist bound EPOR were used [21] for this analysis (Fig. 1A and B). The structure of the protein was corrected.

for missing atoms or unknown units. In addition, all the heteroatoms including water molecules and any co-crystallized solvent were removed from the PDB file to simplify the visualization.

**Fig. 2:**
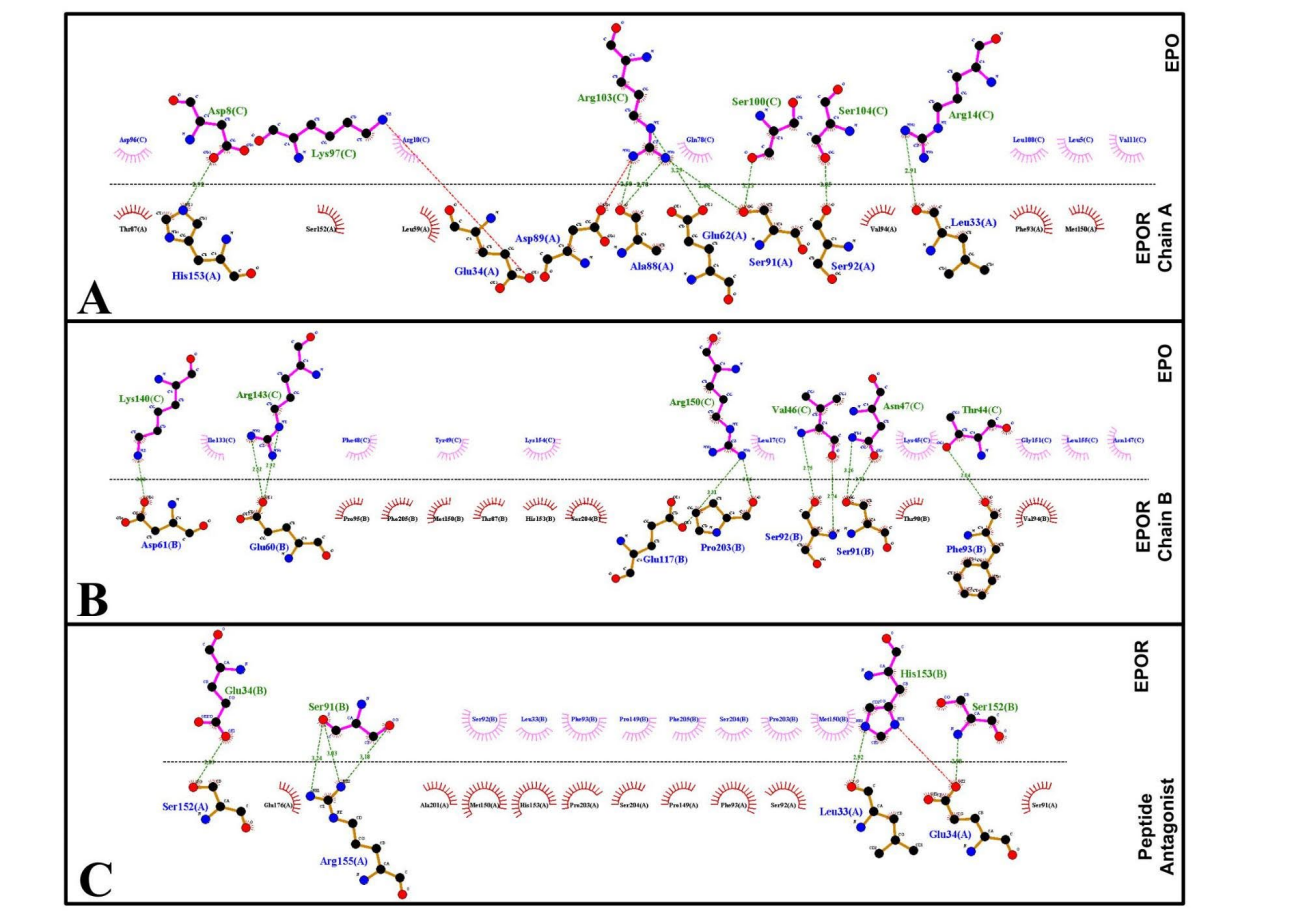
2-dimensional view of the dimer interface of EPOR (amino acid side chain residue stereo view). Chains A and B are shown in first two panels, respectively interacting with EPO. The major clusters of hydrophobic residues in the dimer interface are shown as semicircles with median directed at amino acids/region interacting from other partners. The residues of interchain salt bridges and hydrogen bonds, as determined by DIMPLOT in LIGPLOT, are shown as dashed connections shown as stick models. **A** EPOR (ChainA)-EPO with key EPOR residues highlighted by circles. **B** EPOR (ChainB)-EPO with key EPOR residues highlighted by circles. **C** EPOR monomer interacting with an antagonist

Furthermore, a schematic 2-dimensional representation of the protein-protein complexes DIMPLOT-plots interactions across selected protein or domain interfaces was studied [22] (Fig. 2). The diagrams reflect the patterns of hydrogen-bond interaction and the hydrophobic interactions between the proteins. Additionally, we superpose of related LigPlots to highlight similarities and differences between EPOR chain A and B binding with EPO (Fig. 2A and B, and 2 C).

Upon comparing the binding map of both chains of EPOR with EPO there is surprisingly a 44% overlap spatially even though interaction amino acid identity is only 14% and biochemical conservancy of 29%. This is an example of binding convergence where two peptides bind to the same site, but the interesting thing is both of those distinct peptides are present in a single EPO hormone molecule. The most critical amino acid side chain residues were Ser91 and His153 on EPOR (see red and green circle in the Figure A-C) they were involved in both dimerization and binding of agonists/ antagonists. The distance between both residue side chains is 66Å which is much larger for a drug target site (typically 20Å) also there is the absence of a possible small molecule binding cavity at the site.

The unique 120-degree angular relationship of the two EPOR sub-units as previously reported was clear from visualizing the dimer [23]. This asymmetric homodimerization in presence of EPO instigates the phosphorylation cascade responsible for the activity. As only 5–6% activation is needed for normal signal transduction even a high level of inhibition is insufficient to block this activation [24]. This calls for a knockout strategy to block the phosphorylation cascade.

### Verification of vagus nerve-specific Mpz-EPOR-KO mice

**Fig. 3:**
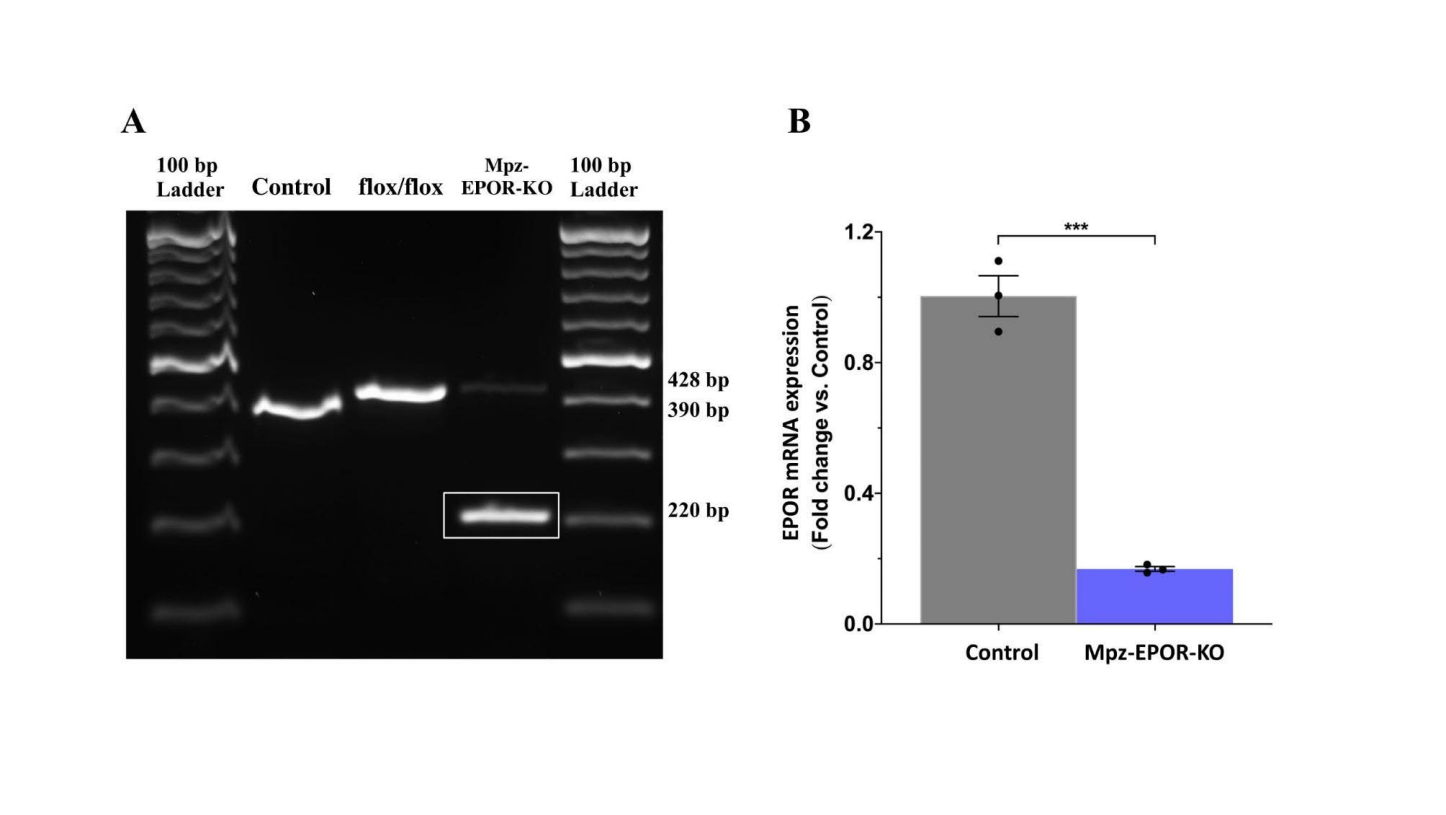
Verification of vagus nerve specific Mpz-EPOR-KO in mice. **A** PCR genotyping with DNA isolated from the vagus nerve revealed the presence of the 220-bp PCR product, resulting from MpzCre mediated recombination in the Schwann cell of MpzCre-EPOR^flox/flox^ mice and not in wild-type (control; 390 bp) and flox/flox (428 bp) mice (n = 3). **B** qRT-PCR analysis confirms a significant knockout of the EPOR gene in the vagus nerve as compared to control mice. Values were normalized to glyceraldehyde-3-phosphate dehydrogenase (GAPDH). Unpaired t-tests and data were expressed as means ± SEM, *** P < 0.0002, Control vs. Mpz-EPOR-KO, n = 3

We have established Schwann cell specific Mpz-EPOR-KO mice using a multi-step breeding process of EPOR^flox/flox^ homozygous and MpzCre hemizygous mice [18]. Briefly, loxP-flanked exons 1–4 of the EPOR genomic sequence were ablated in Schwann cells using the Cre-loxP system, and deletion of EPOR was verified by genotyping of genomic EPOR alleles in VN (Fig. 3A) using oligonucleotide primers (Table 1). The expected size of the PCR product (EPOR band) following amplification of the EPOR gene in wild-type and MpzCre-EPOR^flox/flox^ homozygous mice with floxed is 390 and, 428 bp respectively (Fig. 3A; Table 1). Mutant tissues containing Schwann cells additionally displayed a truncated 220 bp EPOR fragment in the VN, thus confirming Cre-mediated successful deletion of loxP-flanked exons 1–4 in the EPOR gene (Fig. 3A; Table 1). We have also shown interest to see the effective deletion of EPOR at the mRNA level. The quality of extracted total RNA using RNA integrity number (RIN) was 7.2 to 7.8, which is within the normal range to avoid nonspecific gene expression. The qRT-PCR data confirmed a significant knockdown of the EPOR in VN (Fig. 3B; ***P < 0.0002). The primer sequences of EPOR are mentioned in Table 2. However, the expression of small, fainted band (Fig. 3A) and fold change (Fig. 3B) in the VN of the Mpz-EPOR-KO mice might be due to non-neuronal cells EPO-receptors (e.g., fibroblast, immune cells). Thus, we have shown interest in checking the absolute KO of EPOR in VN-derived primary Schwann cells.

**Table 1:** Primer sequences and products for genotyping

Name	Primer sequences	Products
EPOR-1	5’-CTCCAGCCCAGTCCACCAACTGGG-3’	390 bp (WT)
EPOR-2	5’-GCGGGTAGTGGTACAGCACTTGCC-3’	428 bp (floxed)
EPOR-3	5’-CCGTTCTTGGCTCAAAGCCAATC-3’	220 bp (excised)
Cre-F	5’-AGGGATCGCCAGGCGTTTTC-3’	154 bp
Cre-R	5’-CTATATCTTCAGGCGCGCGGTC-3’	

**Table 2:** Primer sequences for qRT-PCR

Name	Primer sequences
EPOR-F	5’-GGGCTCCGAAGAACTTCTGTG-3’
EPOR-R	5’-ATGACTTTCGTGACTCACCCT-3’
GAPDH-F	5’-AGGTCGGTGTGAACGGATTTG-3’
GAPDH-R	5’-TGTAGACCATGTAGTTGAGGTCA-3’

### Verification of vagus nerve Schwann cells specific Mpz-EPOR-KO mice

**Fig. 4:**
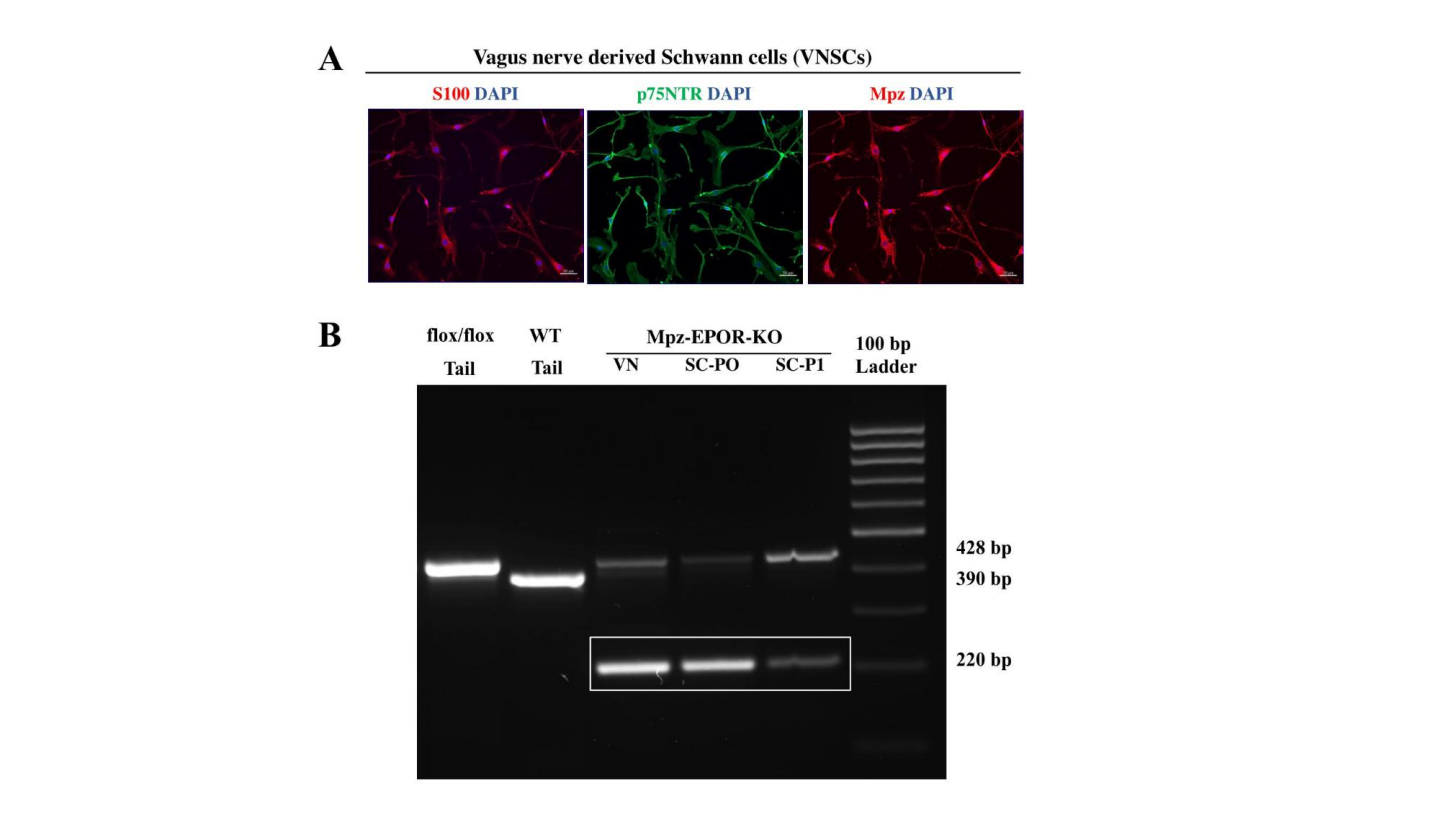
Verification of vagus nerve Schwann cell-specific Mpz-EPOR-KO in mice. **A** Characterization of mouse vagus nerve derived Schwann cells (VNSCs). The identity and purity of SCs were confirmed using IF staining of S100, p75NTR, and Mpz under a fluorescent microscope (ZEISS Apotome 2). The purity of the cultured SCs (99%) was analyzed by double positive staining of DAPI with S100/p75NTR/Mpz markers from 3 independent experiments. Each image represents 3 images from 3 independent experiments. Scale bar: 50 μm, n = 3. **B** PCR genotyping with DNA isolated from the vagus nerve and VNSCs (passage zero, SC-P0; passage one, SC-P1) revealed the presence of the 220-bp PCR product, resulting from MpzCre mediated recombination in the Schwann cell of MpzCre-EPOR^flox/flox^ mice and not in wild-type (control; 390 bp) and flox/flox (428 bp) mice (n = 3)

To confirm Schwann cell-specific Mpz-EPOR-KO, we aimed to isolate SCs from the vagus nerve. The identity and purity of SCs were confirmed (99%) using three markers (S100, p75NTR, and Mpz) by IF staining (Fig. 4A). The genotyping data (Fig. 4B) shows absolute KO of EPOR in the purified SCs (see 220 bp vs. 428 bp; passage zero, SC-PO) as compared to VN. However, interestingly, passage one of SCs (SC-P1) shows reduced EPOR-KO, and this might be due to the transition of SCs (passage 0 to 1) from myelin to non-myelin status. Tail samples of the wild type and EPOR^flox/flox^ served as a control. Thus, our data confirm the complete knockout of EPOR in SCs, which is associated with the myelin status of cells.

### Verification of enteric glial cells specific Mpz-EPOR-KO mice

**Fig. 5:**
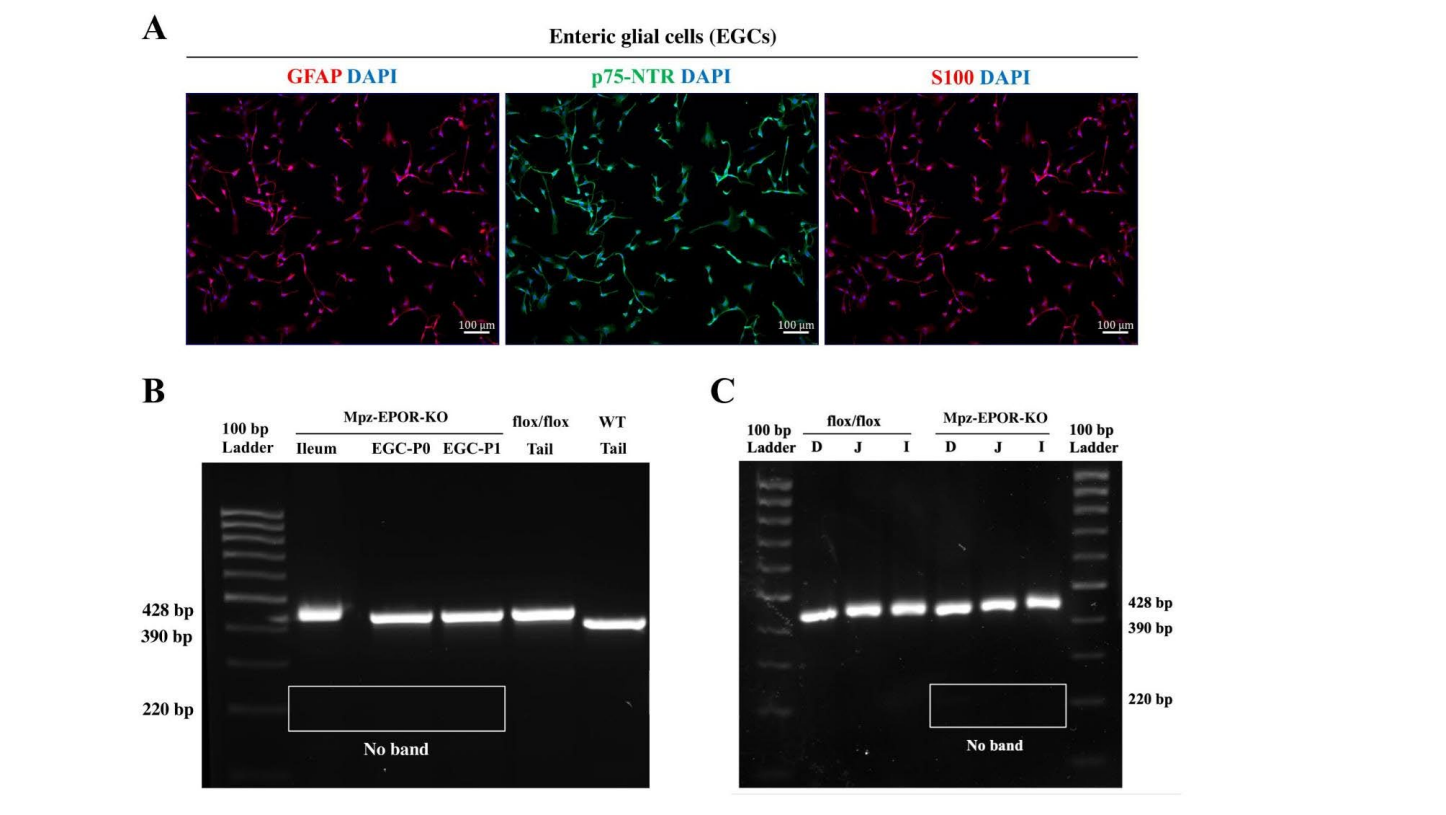
Verification of enteric glial cell-specific Mpz-EPOR-KO in mice. **A** Characterization of mouse enteric glial cells (EGCs). The identity and purity of SCs were confirmed using IF staining of GFAP, p75NTR, and S100 under a fluorescent microscope (ZEISS Apotome 2). The purity of the cultured EGCs (99%) was analyzed by double positive staining of DAPI with GFAP/p75NTR/S100 markers from 3 independent experiments. Each image represents 3 images from 3 independent experiments. Scale bar: 100 μm, n = 3. **B** PCR genotyping with DNA isolated from EGCs (passage zero, EGC-P0; passage one, EGC-P1) revealed no deletion of EPOR in MpzCre-EPOR^flox/flox^ mice. EPOR bands of wild-type (control; 390 bp) and flox/flox (428 bp) tail samples of mice are depicted (n = 3). **C** PCR genotyping with DNA isolated from the segments of the intestine (D, duodenum; J, jejunum; I, ileum) revealed no deletion of EPOR in MpzCre-EPOR^flox/flox^ mice (n = 3)

Following EPOR-KO confirmation in VNSCs, next, we aimed to check the status of EPOR-KO in enteric glial cells (EGCs), which are associated with the cell bodies and processes of enteric neurons throughout the digestive tract [25]. To verify EPOR deletion, we have isolated EGCs and the identity and purity of EGCs were confirmed (99%) using S100, p75NTR, and GFAP by IF staining (Fig. 5A). The genotyping data shows no deletion of EPOR in the purified EGCs (Fig. 5B), both in the passage “0” and “1” (see 220 bp; passage zero, EGC-PO; passage one, EGC-P1) like ileum segment. Tail samples of the wild type and EPOR^flox/flox^ served as EPOR controls. This was also true in all three segments of the intestine (D, duodenum; J, jejunum; I, ileum) (Fig. 5C). In a nutshell, our data shows that EPOR knockout was specific to Mpz-associated Schwann cells, which is not true for the intestine or intestinal glial cells.

### Effect of erythropoietin treatment on postoperative intestinal transit time

**Fig. 6:**
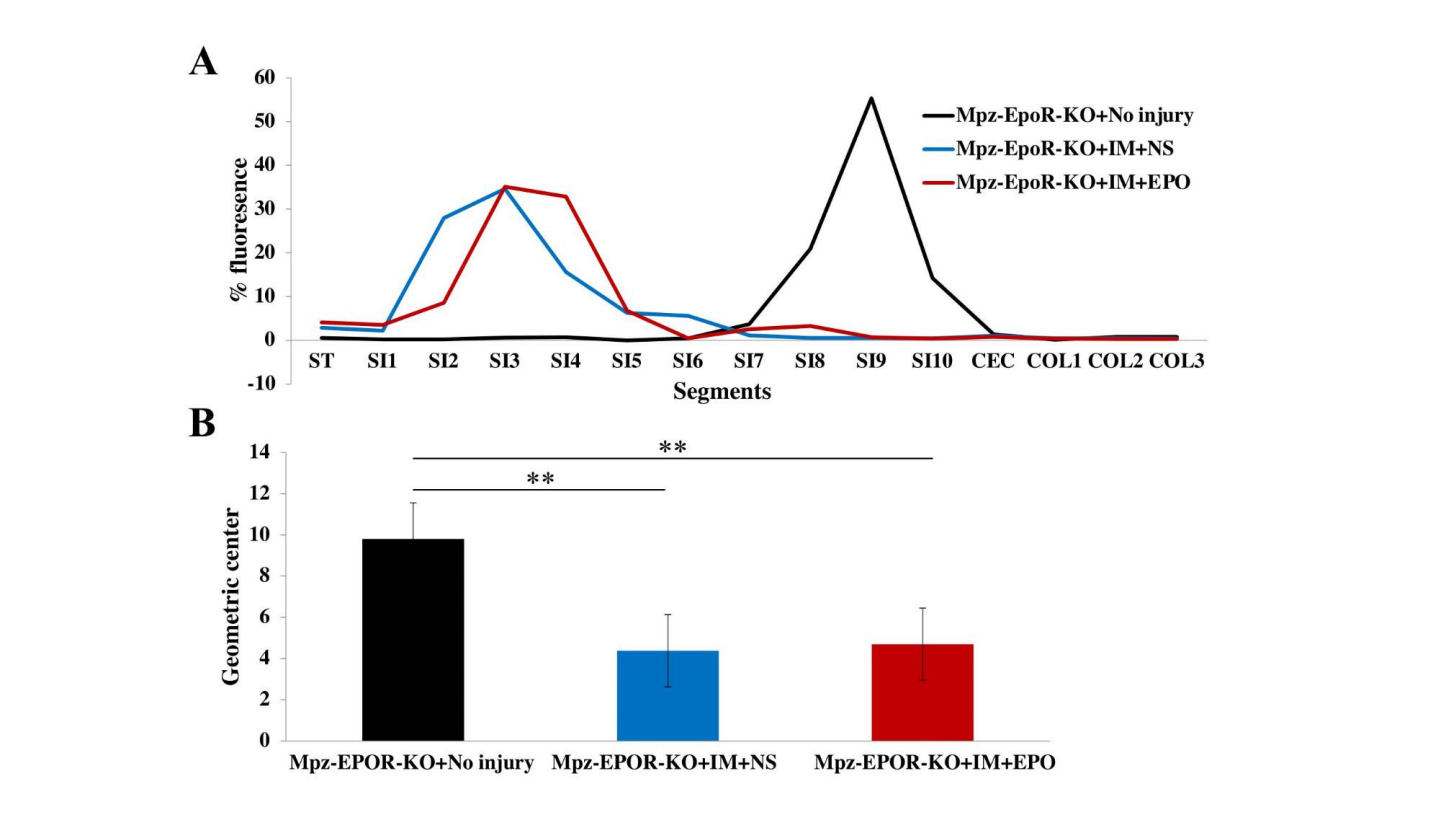
Role of EPO on intestinal transit time following IM. **A** The percent fluorescence of the stomach (ST), ten segments of the small intestine (SI 1–10), cecum (CEC), and three segments of the colon (COL 1–3). **B** Gastrointestinal transit geometric center. Black graph (control, no manipulation), blue graph (IM + saline), and red graph (IM + EPO) treatment group. One-way ANOVA, Tukey’s multiple comparisons test. Data were expressed as means ± SEM, **P < 0.0021, No injury vs. saline vs. sham; n = 4 /group. NS, Normal Saline

We aimed to determine the effect of EPO treatment on the gastrointestinal motility of Mpz-EPOR-KO mice after IM. ITT, a method to assess gastrointestinal transit, was evaluated in controls (no manipulation) and intestine-manipulated animals 24 h after surgery, as mentioned in our established protocol [11]. Figure 6 illustrates the functional motility of the intestine after measuring the percent concentration of fluorescence FITC-labeled dextran in the bowel of animals (n = 4/ group). Intestinal manipulation (IM + saline; blue line; n = 4) significantly affected ITT compared to the control group (black line; n = 4), where fluorescence was abundant in the proximal part of the small intestine (Fig. 6A), which showed a reduction in the mean geometric center (Fig. 6B, **P < 0.0021). Inquisitively, EPO treatment (red line; n = 4) did not improve IM-induced delay with no effective distribution of fluorescence in the small intestine (Fig. 6A) or reversal of the mean geometric center (Fig. 6B; **P < 0.0021) to control level. There was no difference between the saline and EPO treatment groups. However, our previous study has shown that EPO significantly improved ITT in the setting of POI using wild-type mice [11]. This potentially could show the conditional role of VNSCs EPOR in the improvement of ITT following POI. In summary, we can conclude that EPO treatment might benefit ITT through direct effect of EPOR on the VNSCs rather than EGCs.

## Discussion

We have generated a novel Schwann cell-specific conditional knockout EPOR mouse model. Our results provided direct evidence that vagus nerve Schwann cells specific EPOR plays an obligatory role in EPO-induced improvement in intestinal motility following POI. Here, EPO treatment has not shown a beneficial effect on ITT compared to the saline group. However, our previous study has shown a significant increase in ITT following EPO treatment in the setting of the POI-wildtype mouse model, where EPOR was intact in VN Schwann cells [11]. EPO treatment improves POI functional recovery via mediating Vagal nerve EPOR signaling. We have supported this by conditional knockout of Schwann cells specific-EPOR at both the DNA and mRNA levels using PCR and qRT-PCR, respectively.

Next, we set a confirmatory experiment to see the status of the EPOR in cultured SCs (S100/p75NTR/Mpz positive) of Mpz-EPOR-KO mice. In vitro, SCs studies are applicable to elucidate EPO molecular signaling under different translationally relevant stressor conditions. Our result shows the absolute knockout of EPOR in passage zero, VNSCs. Interestingly, we have observed partial deletion of EPOR in passage one of the VNSCs, which might be due to a shift in the myeline status of the cells. Thus, our data precisely confirms EPOR deletion was absolute with myelinated Schwann cells but not with the non-myeline cells that include neuronal fibroblasts, endothelial cells, and immune cells.

In parallel, we have shown our further interest in characterizing Mpz-EPOR-KO mice, where we aimed to determine the status of EPOR-KO in the intestine that is innervated and regulated by VN. Genotyping data shows no deletion of EPOR in all the intestinal segments. Inquisitively, this was also true with EGCs. In summary, the development of the EPOR-KO condition mice was specific to neuronal-glial cells (VNSCs) rather than intestinal glial cells (EGCs). The absence of the functional improvement in POI with EPO treatment in the KO mice, speaks for the role of vagus nerve EPOR in mediating intestinal recovery.

In conclusion, using vagus nerve Schwann cells-specific Mpz-EPOR-KO mice, our preliminary findings indicate that EPO-EPOR coupling plays an obligatory role in exogenous EPO-induced functional recovery following POI. Further characterization of this novel Mpz-EPOR-KO mouse model and investigations of EPO-induced downstream signaling pathways concerning EPOR expression, paracellular communications, the severity of POI, EPO dosages, and timing of EPO administration could provide remarkable insights into clinical translation use.

## Methods

### In-silico bioinformatics analysis

UCSF chimera software was used to create a schematic visualization of EPOR activation by EPO-mediated dimerization [26]. The X-ray crystal structures of EPOR bound to EPO (PDB ID: 1CN4) and antagonist-bound EPOR (PDB ID: 1ERN) were downloaded from RCSB Database [21]. The structure of the protein was corrected for missing atoms or unknown units using the MVD (Molegro Virtual Docker) program. In addition, all the heteroatoms including water molecules and any co-crystallized solvent were removed from the PDB file to simplify the visualization. To visualize the binding of EPO and inhibitor with EPOR dimerization site, we used LigPlot+, a graphical front-end to the LigPlot and DimPlot programs (http://www.ebi.ac.uk/thorntonsrv/software/LigPlus/). DIMPLOT produced a schematic 2-D representation of the protein-protein complexes DIMPLOT-plots interactions across selected protein or domain interfaces [22]. The diagrams reflect the patterns of hydrogen-bond interaction and the hydrophobic interactions between the proteins. Additionally, we superpose related LigPlots to highlight similarities and differences between EPOR chain A and B binding with EPO (Fig. 2). The 3D representation of the same interaction was viewed in VMD software [27]. In Fig. 1 colors are used to encode different properties of the contacts between amino acids (colored dots in the plots).

### Animals

The experimental design and animal protocols were approved by the Institutional Animal Care and Use Committee (IACUC) at The Pennsylvania State University College of Medicine, Hershey, PA. Twelve- to 16-week-old male mice weighing 25 to 30 g were used.

### Generation of mice with conditional knockout of EPOR in Schwann cells

The specific knockout of EPOR in Schwann cells (MpzCre-EPOR^flox/flox^, synonym with Mpz-EPOR-KO) was generated by cross-mating floxed EPOR (EPOR^flox/flox^) mice with myelin protein zero (Mpz) promoter-driven Cre recombinase (MpzCre) mice from Jackson Laboratories as described in our publication [18]. EPOR deletion was confirmed using polymerase chain reaction (PCR) with genomic DNA from the tail, intestine segments (duodenum, jejunum, and ileum), vagus nerve, and vagus nerve derived Schwann cells (VNSC). Primers were purchased from Invitrogen (Life Technologies) and the sequences of primers are listed in Table 1.

### Animal euthanasia

Experiment animals were euthanized using an overdose (5%) of isoflurane in a sterile condition of drop jar method followed by cervical dislocation, where the depth of the anesthesia was confirmed using respiration status and the pedal reflex.

### RNA extraction, cDNA synthesis, and quantitative reverse transcription PCR

To assess EPOR gene expression, the vagus nerve of the MpzCre-EPOR^flox/flox^ and EPOR^flox/flox^ (control) mice were harvested and the total RNA was extracted using miRNeasy mini kit (#217,004; Qiagen) [18]. Next, RNA was reverse transcribed to cDNA (#1,708,840; Bio-Rad), and the relative mRNA expression of the EPOR (normalized to GAPDH) was quantified using Fast SYBR Green Master Mix (#4,385,612; Applied Biosystems). Invitrogen (Life Technologies) primer sequences are listed in Table 2. The quality of the RNA was evaluated using the RNA integrity number (RIN) of Agilent Bioanalyzer 2100 (Agilent Technologies; Germany) [17].

### Isolation, culture, and characterization of vagus nerve Schwann cells

Mouse vagus nerve-derived Schwann-cell (VNSC) isolation and ex vivo expansion was performed as described in the previous studies [17, 28]. Briefly, after euthanizing mice, the neck area of the mouse was shaved and swabbed with 70% ethanol. A midline cervical incision was made, exposing the salivary glands and trachea. Subcutaneous tissues between the sternomastoid and sternohyoid muscles along the trachea were separated using blunt dissection revealing the common carotid artery and the cervical vagus nerve. VN and the carotid artery are located parallel to each other and were separated using forceps to harvest ≈ 2 cm of nerve, which was placed in a Petri dish containing an ice-cold DMEM basal medium (#11,995,073; Gibco™). Nerve fibers were dissociated using a mechanical (forceps) and enzymatic [0.2% collagenase type I (#17,018,029; Gibco™): 0.2% dispase II (#D4693; Millipore Sigma) in a DMEM basal media] method. The digested cell suspension was centrifuged at 250 x g for 5 min and resuspended in a DMEM medium (DMEM supplemented with 10% (vol/vol) FBS, 1% (vol/vol) streptomycin/penicillin) for plating in PLL (poly-L-lysine) (#P4707; Millipore Sigma) coated T25-flask. After overnight incubation, actively dividing fibroblast cells were killed using 10µM cytosine β-D-arabinofuranoside hydrochloride (#C6645; Millipore Sigma) for 24 h. Then, cells were incubated and maintained with mouse Schwann-cell medium [(#M1700-57; ScienCellTM; supplemented with 5% (vol/vol) FBS, 1% (vol/vol) streptomycin/penicillin, and 1% (vol/vol) Schwann cells growth factor)]. Passage 1 cells were used for IF analysis using S100 (1:100; #MA5-12969; Invitrogen), p75NTR (1:250; #AB1554; Sigma-Aldrich), and Mpz (1:1000; #PZO; Aves Labs) followed by incubation with the appropriate secondary antibody Alexa Fluor 594 (1:500; #A11032; Invitrogen), Alexa Fluor 488 (1:500; #A11008; Invitrogen), and Alexa Fluor 647 (1:500; #A21449; Invitrogen).

### Isolation, culture, and characterization of enteric glial cells

Mouse enteric glial cell isolation and ex vivo expansion was performed as previously described with slight modification [29]. Briefly, after mouse preparation, the skin and abdominal muscles were dissected using a microscope to expose the intestine (duodenum, jejunum, and ileum). Care was taken to avoid bleeding, fat tissues, and mesentery while harvesting the intestine. Next, the contents of the intestinal lumen were flushed out using ice-cold 1XDPBS with repeated wash. Intestine tissue is secured firmly by inserting a wet wooden stick into the intestinal lumen and later wetted cotton tip was gently rubbed along the length of the intestine to harvest the myenteric plexus. The digestion of the myenteric plexus and culturing of enteric glial cells procedure was like Schwann cell isolation methods. The purity of EGC was confirmed using IF staining of S100 (1:100; #MA5-12969; Invitrogen), p75NTR (1:250; #AB1554; Sigma-Aldrich), and GFAP (1:1000; # 13–0300; Invitrogen) followed by incubation with the appropriate secondary antibody Alexa Fluor 594 (1:500; #A11032; Invitrogen), Alexa Fluor 488 (1:500; #A11008; Invitrogen), and Alexa Fluor 647 (1:500; #A21449; Invitrogen).

### Experimental design and animal model of postoperative ileus (POI)

Eighteen mice were randomly allocated into three groups. Group 1 animals were normal with no surgical manipulation (n = 4). Groups 2 and 3 animals underwent intestinal manipulation (IM) and these mice received intraperitoneal injections of either normal saline (0.1 mL/mouse, Group 2, n = 4) or EPO (5000 IU/kg; Epoetin alfa, PROCRIT®, Group 3) immediately after surgery. IM was carried out as described in our previous publication [11]. No manipulation of the intestine and manipulated saline-injected animals served as controls. Briefly, animals were anesthetized using 3% isoflurane inhalation and maintained with 1.5% until completion of surgery. A midline incision was made to expose the peritoneal cavity and the small bowel was eviscerated onto moist gauze and gently manipulated between two moist cotton applicators. IM was repeated three times for each animal during the procedure and was performed by a single-blinded surgeon to ensure uniformity in the injury and to avoid variability among surgeons. After IM, abdominal muscles were closed using interrupted sterile non-absorbable surgical sutures (5/0, Ethicon, Norderstedt, Germany). The skin was closed with surgical staples and no injury was severe enough to cause bleeding or mortality. The mice were not injected with pain medication following surgery to avoid interference with intestinal motility.

### Functional studies

Twenty-four hours after IM, in vivo gastrointestinal transit study was performed as described in our protocol [11]. Briefly, intestinal transit time (ITT) was measured in all animals/ groups at 24 h postoperatively by evaluating the intestinal distribution of fluorescence FITC-labeled dextran (70,000 MW; #46,945; Sigma-Aldrich). All the animals were gavaged with FITC-labeled dextran (10 µl of 25 mg/ml stock solution) for ninety minutes and later sacrificed by an overdose of isoflurane followed by cervical dislocation. The contents of the stomach, small intestine (10 equal segments), cecum, and colon (3 equal segments), were quantified for FITC using a spectrophotometer (Ascent; Lab system Inc.) at 488 nm. The percentage of ITT and geometric center (GC) of distribution [Σ (percent of total fluorescent signal in each segment multiplied by the segment number)/ 100 for quantitative comparison among experimental groups] was quantified using FITC-Dextran.

### Statistical analysis

All data were analyzed using GraphPad Prism Version 8.4.3 (San Diego, USA). Comparisons between three groups with n ≥ 4 were performed via ordinary one-way analysis of variance (ANOVA), and Tukey’s multiple comparisons test after confirmation of normally distributed data. For comparisons of two groups, (n per group ≥ 3), two-tailed, unpaired t-tests were used. All values are presented as means ± SEM. Significance levels (P values < 0.05) were documented using standard symbols (**, and *** correspond to P < 0.0021, P < 0.0002, respectively).

## Electronic supplementary material

Below is the link to the electronic supplementary material.

Supplementary Material 1

## Data Availability

Correspondence and requests for materials should be addressed to Manmeet Rawat and Walaa Elfar.

## List of Abbreviations

KO: Knock out
EGC: Enteric glial cells
POI: Postoperative ileus
IM: Intestinal manipulation
VNSCs: Vagus nerve derived Schwann cells
FITC: Fluorescein isothiocyanate
GFAP: Glial fibrillary acidic protein
p75NTR: p75 neurotrophin receptor
Mpz: Myelin protein zero
EPO: Erythropoietin
EPOR: Erythropoietin receptor
GAPDH: glyceraldehyde 3-phosphate dehydrogenase
RIN: RNA integrity number
ITT: Intestinal transit time
GC: Geometric center
FBS: Fetal bovine serum
DMEM: Dulbecco’s modified eagle medium
DPBS: Dulbecco’s phosphate-buffered saline
IF: Immune fluorescence.
