# Supplementary material for: A critical role for erythropoietin on vagus nerve Schwann cells in intestinal motility
Source: BMC Biotechnol. 2023 May 1;23:12. doi: 10.1186/s12896-023-00781-x (PMC10152589; doi:10.1186/s12896-023-00781-x)
Supplement: Supplementary file 1 — Supplementary Material 1 [file 12896_2023_781_MOESM1_ESM.pdf]

Additional file 1: Uncropped PCR genotyping images

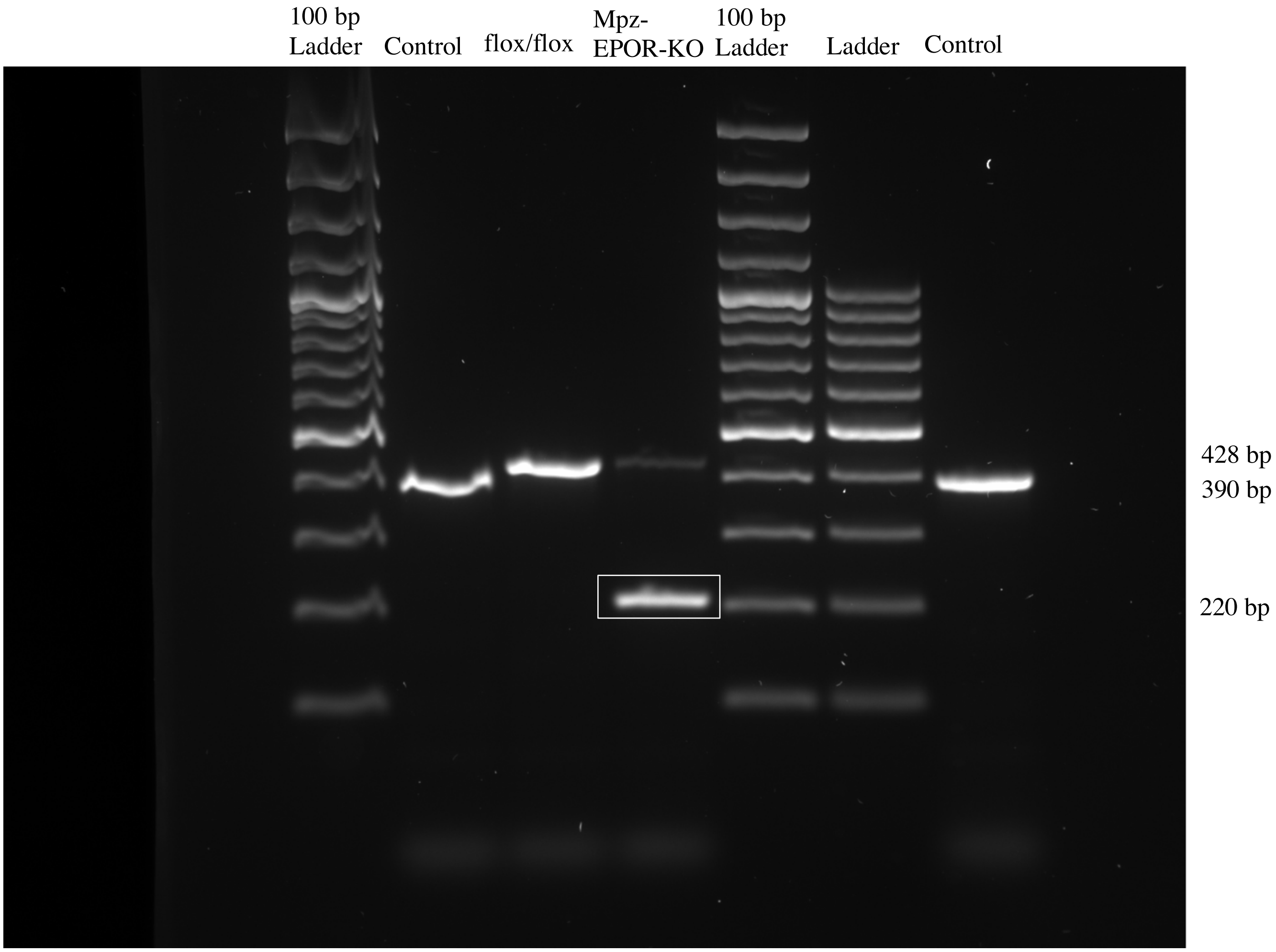

Figure 3A. Verification of vagus nerve specific Mpz-EPOR-KO in mice.

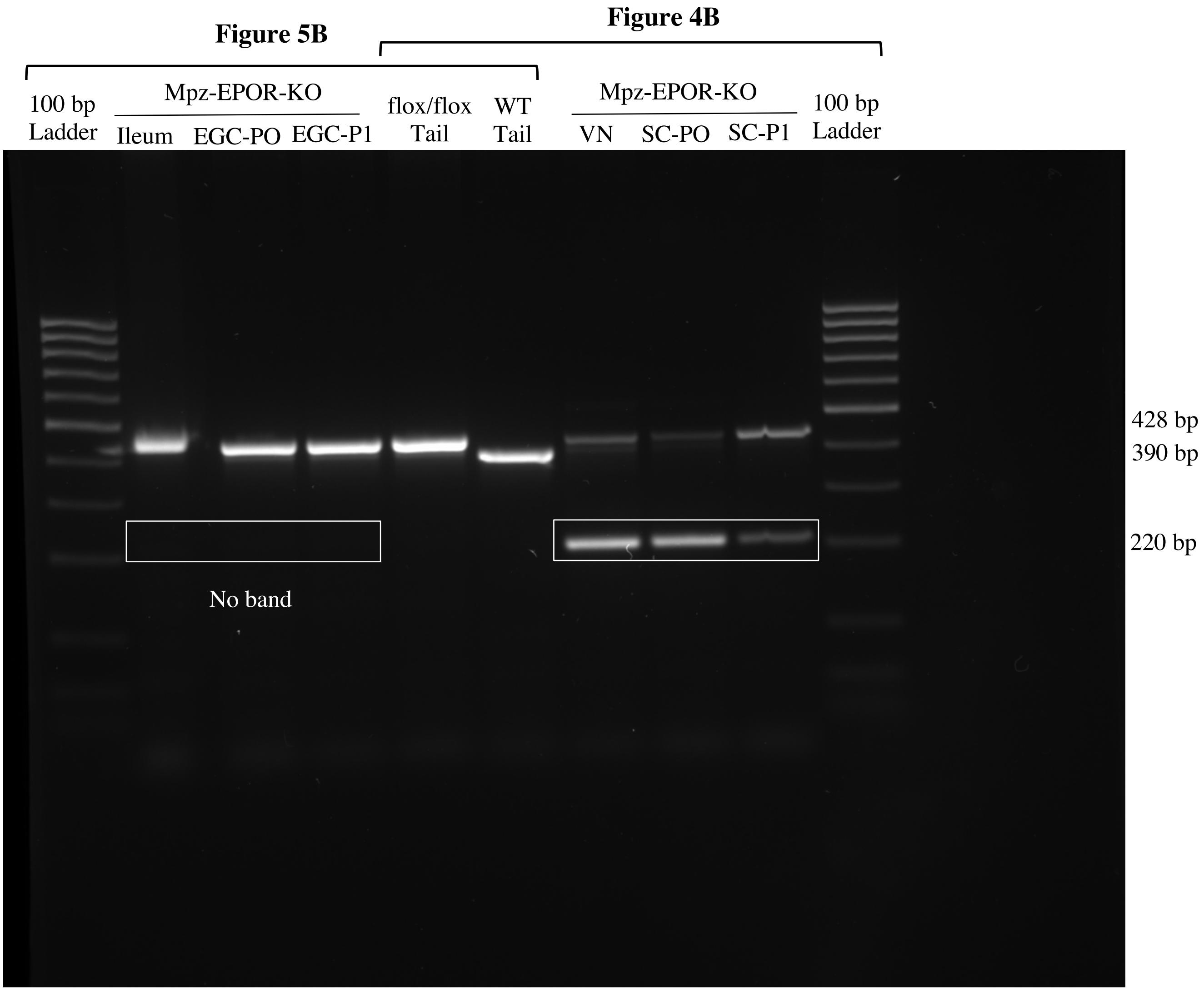

Figure 4B. PCR genotyping with DNA isolated from the vagus nerve and VNSCs (passage zero, SC-P0; passage one, SC-P1).

Figure 5B. PCR genotyping with DNA isolated from ileum and EGCs (passage zero, EGC-P0; passage one, EGC-P1).

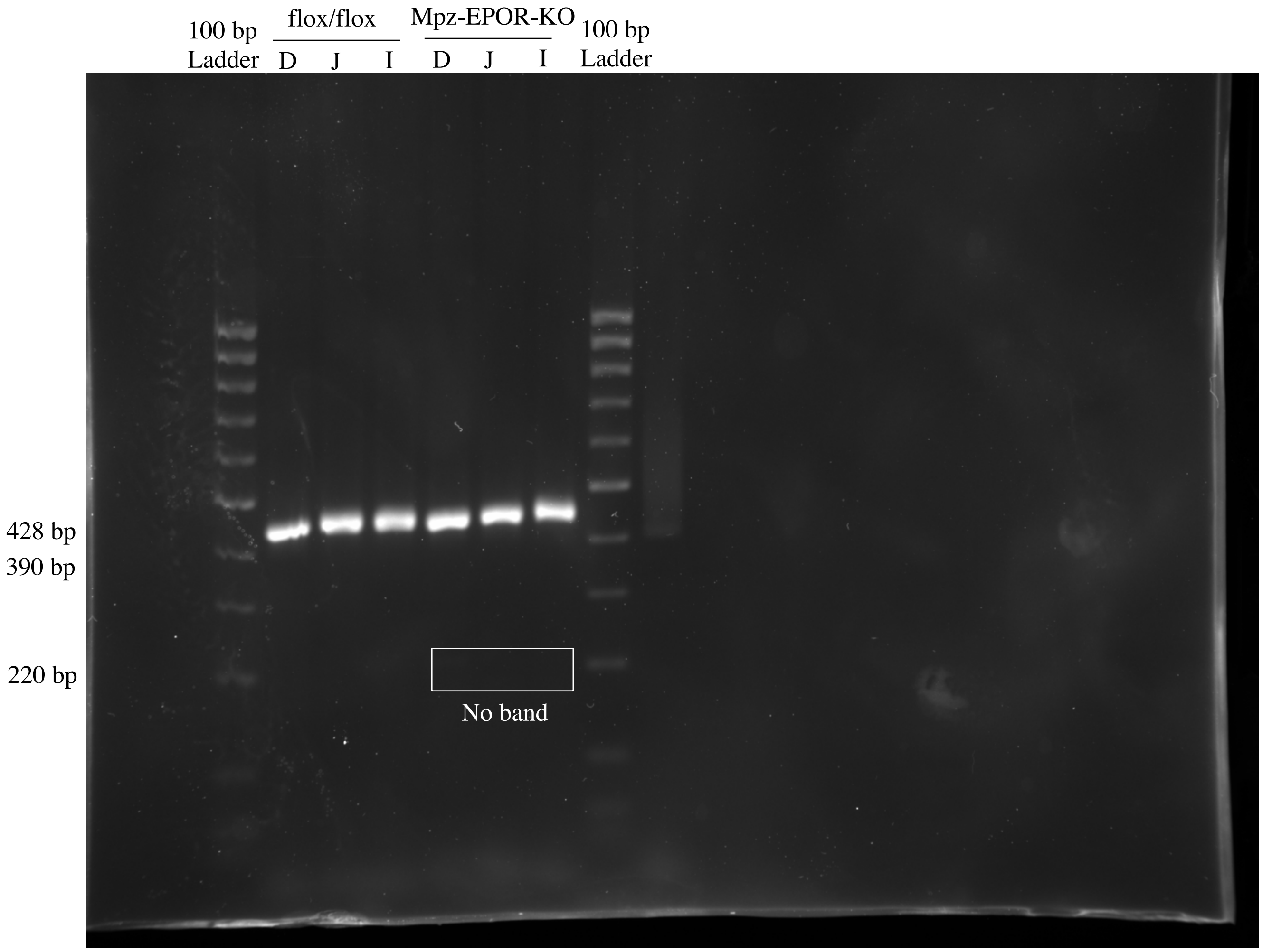

Figure 5C. PCR genotyping with DNA isolated from the segments of the intestine (D, duodenum; J, jejunum; I, ileum).
